# Adverse Outcome Pathway‐Informed Integrated Testing to Identify Chemicals Causing Genotoxicity Through Oxidative DNA Damage: Case Study on 4‐Nitroquinoline 1‐Oxide

**DOI:** 10.1002/em.70011

**Published:** 2025-05-08

**Authors:** Elizabeth Huliganga, Eunnara Cho, Carol D. Swartz, Andrew Williams, Leslie Recio, Jesse J. Salk, Francesco Marchetti, Carole L. Yauk

**Affiliations:** ^1^ Department of Biology University of Ottawa Ottawa Canada; ^2^ Environmental Health Science and Research Bureau, Health Canada Ottawa Canada; ^3^ Inotiv RTP Morrisville North Carolina USA; ^4^ Scitovation Durham North Carolina USA; ^5^ Division of Hematology and Oncology University of Washington School of Medicine Seattle WA USA; ^6^ Department of Biology Carleton University Ottawa Canada

**Keywords:** adverse outcome pathway, duplex sequencing, error‐corrected sequencing, genetic toxicology, in vitro toxicology, new approach methodologies

## Abstract

Adverse outcome pathways (AOPs) provide a framework to organize and weigh evidence linking molecular interactions of toxicants in cells to adverse outcomes relevant to risk assessment or regulatory decision‐making. Applying this framework facilitates the interpretation of data produced using new test methods. We used an existing AOP (AOP #296) that describes how oxidative DNA damage leads to mutations and chromosomal aberrations to develop an integrated testing strategy to evaluate whether a chemical operates through this pathway. We exposed human TK6 cells to increasing concentrations of 4‐nitroquinoline 1‐oxide (4NQO), a tobacco mimetic that causes oxidative DNA damage, in a time‐series design. We measured oxidative DNA damage and strand breaks using the high‐throughput CometChip assay with and without formamidopyrimidine DNA glycosylase (Fpg), alongside analyses of micronucleus (MN) frequency by flow cytometry, and mutations by error‐corrected sequencing (duplex sequencing—DS). Our analysis shows how these methods can be combined to quantify 4NQO‐induced, concentration‐ and time‐dependent increases in: (a) oxidative DNA damage (occurred early and at low concentrations); (b) strand breaks (remained elevated to 6 h post‐exposure); (c) MN frequency (at 24 h); (d) mutation frequency (at 48 h); and (e) C > A transversions consistent with expected substitutions induced by oxidative DNA lesions. The time series shows the repair of oxidative DNA damage with persistent strand breaks remaining at 6 h. Overall, we provide an example of an AOP‐informed testing strategy and contribute to the quantitative understanding of AOP #296. We also demonstrate the value of DS as an effective approach for mutagenicity assessment.

## 1 Introduction

Chemical genotoxicity can lead to numerous adverse outcomes including heritable genetic disease, cancer, and accelerated aging. Thus, human health risk assessment includes careful evaluation of a chemical's potential to cause DNA damage that can lead to mutations, chromosomal aberrations, or aneuploidy. Standard genotoxicity testing has historically relied on assays such as the Ames test to qualitatively classify chemicals as genotoxic hazards. Depending on the regulatory context (i.e., the country or regulatory body; or the context of chemical use—pharmaceutical (Galloway 2017), cosmetic (Speit 2009), or industrial (Ji et al. 2017)), a positive in vitro genotoxicity test might then require an in vivo rodent follow‐up such as the micronucleus (MN) or the transgenic rodent mutation assays. The standard in vitro assays are hampered by biologically irrelevant positive results, while the follow‐up in vivo assays require considerable resources and time, and provide limited insight into mechanisms of action (Dearfield et al. 2017). However, they are currently necessary as the genotoxicity data generated through these standard tests are critical components of the regulatory toolbox for protecting human health.

Given the large numbers of new and existing chemicals awaiting evaluation (Krewski et al. 2019), it is not feasible to apply time and resource‐intensive in vivo testing to determine the genotoxicity for all chemicals of interest. Instead, there is a need to develop in vitro methods that provide mechanistic information and allow the screening and prioritization of chemicals for their potential to cause genotoxicity. Moreover, regulatory agencies are increasingly emphasizing the need to reduce and replace animal testing. Thus, effective, mechanism‐based testing in nonanimal models that accurately predicts endogenous human genetic effects is required. Such a modernized testing strategy should be more cost‐effective and provide improved prediction of in vivo outcomes than current methods. Implementation of a modernized strategy requires the identification and development of a pragmatic interpretation framework with which to use mechanistic information to predict adverse genetic effects (Ankley et al. 2010; Sakuratani et al. 2018; Sasaki et al. 2019).

One tool that can be used to interpret mechanistic data to evaluate mode of action, develop test paradigms, integrate emerging methods, and predict potential in vivo effects, is the adverse outcome pathway (AOP) Framework (Ankley et al. 2010; OECD 2022). The components of an AOP are measurable biological events, called key events (KEs), and the causal relationships between them, called key event relationships (KERs). The first KE is the initial interaction between a chemical and a biomolecule, called the molecular initiating event (MIE), and the last KE is an endpoint considered “relevant to risk assessment or regulatory decision‐making,” called an adverse outcome (AO) (OECD 2022). Each KE describes new and existing test methods that can be used to measure the KE. The KERs describe the biological understanding and supporting empirical evidence that the KEs are causally connected. This enables the use of data from new test methods that capture effects at lower levels of biological organization (e.g., molecular and cellular levels) to be used in predicting downstream effects at higher levels of biological organization. A variety of AOPs have been developed in the area of genetic toxicology to promote the use of emerging methods within the regulatory community (https://aopwiki.org/aops/15; https://aopwiki.org/aops/106; https://aopwiki.org/aops/272) (Chauhan et al. 2021; Marchetti et al. 2016; Yauk et al. 2015).

**FIGURE 1:**
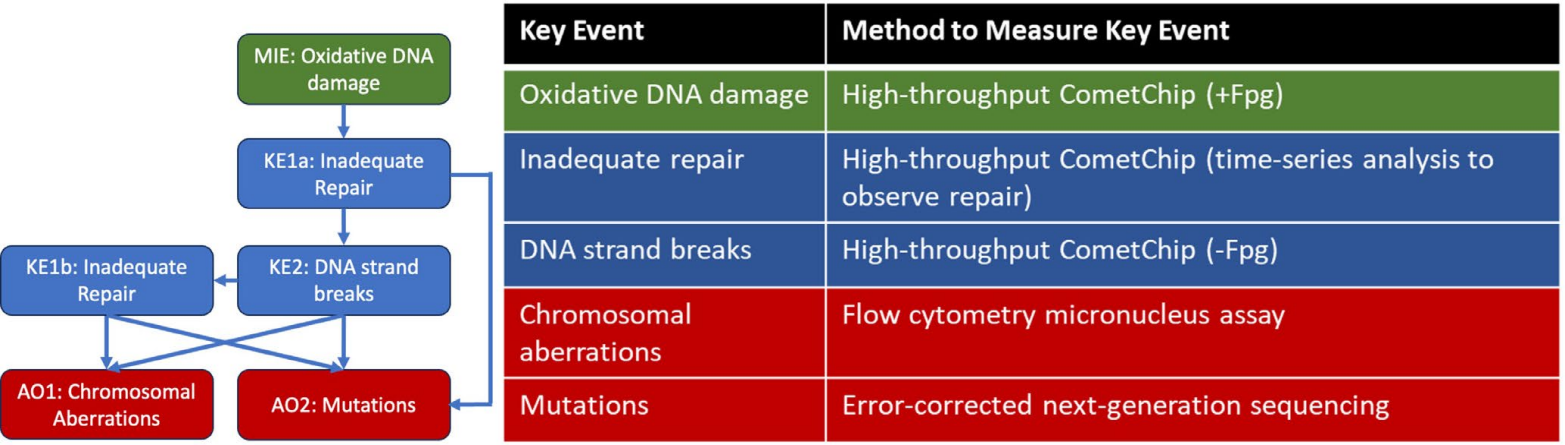
Flow diagram of adverse outcome pathway 296 (AOP 296) “Oxidative DNA damage leading to mutations and chromosomal aberrations” (modified from Cho et al. 2022). The molecular initiating event is shown in green, the key events in blue, and the adverse outcomes in red. The accompanying table shows the methods that were used to measure each of the key events in this study.

An important consideration in genetic toxicology evaluation is whether chemicals operate through direct or indirect genotoxic mechanisms. A central indirect mechanism associated with genotoxicity is the production of reactive oxygen species (ROS) that can cause oxidative DNA damage. AOP #296 describes how oxidative DNA damage leads to chromosomal aberrations and mutations (https://aopwiki.org/aops/296 ) (Cho et al. 2022; Sakuratani et al. 2018) (Figure 1). This AOP begins with an increase in oxidative damage to the nitrogenous bases of DNA (MIE) that overwhelms the repair capacity, leading to inadequate repair of the damage (KE1a). Inadequate repair of the oxidative DNA damage (KE1a) then branches into two paths. It can directly lead to an increase in mutations (AO1) arising from the replication of damaged template DNA and to an increase in DNA strand breaks (KE2) occurring from failed DNA repair processes. Inadequate repair of DNA strand breaks (KE1b) can lead to an increase in chromosomal aberrations (AO2) or mutations (AO1) downstream (Cho et al. 2022). AOP #296 provides a framework for the development of novel integrated testing strategies that can be used for next‐generation genotoxicity testing to determine if a chemical induces DNA damage indirectly through the generation of ROS.

A limitation of AOP #296 is that despite strong biological plausibility and moderate empirical evidence in support of its KERs, quantitative understanding of the AOP is low (Cho et al. 2022). Specifically, there is a lack of understanding of what level of oxidative DNA damage must occur before there is progression to the later KEs or AOs. This is due to a lack of studies that use multiple stressor concentrations and time points within a single experimental design (i.e., to quantify the dose, incidence, and temporal relationships within a specific model system), which is required to build quantitative understanding. Strengthening the base of quantitative evidence for AOP #296 would allow for better use of oxidative DNA damage and DNA strand break measurements to predict chromosomal aberrations and mutations.

The overarching objective of the present study is to apply AOP #296 as an organizational construct on which to develop an integrated test strategy to interpret data from genotoxicity‐testing NAMs (Figure 1). We undertake a case study using human TK6 cells, a routinely used model in genotoxicity testing, and the prototype compound 4‐Nitroquinoline 1‐oxide (4NQO). 4NQO is a carcinogen that is commonly used to study the development and progression of oral squamous cell carcinoma, which is strongly associated with tobacco use (Kanojia and Vaidya 2006). It is metabolized to reactive intermediates that contribute to oxidative stress via redox cycling (Arima et al. 2006; Brüsehafer et al. 2016). This process can lead to both bulky adduct formation and oxidative DNA lesions. 4NQO exposure increases nitric oxide levels (Tan et al. 2020) and total cellular ROS (Abraham et al. 2007), and depletes glutathione (GSH) via 4NQO‐GSH conjugation (Stanley and Benson 1988), leading to an increase in oxidative DNA lesions, predominantly 8‐oxo‐2′‐deoxyguanosine (8‐oxodG) (Arima et al. 2006). Thus, 4NQO serves as a useful model to study both direct DNA damage via adduct formation and oxidative stress‐induced damage. Here, 4NQO‐treated cells were analyzed using modern, quantitative, in vitro genetic toxicology assays : the high‐throughput CometChip assay (Ge et al. 2021; Sykora et al. 2018) and the flow cytometry MN assay (Avlasevich et al. 2006; Bryce et al. 2010).

A second major objective is to provide proof of concept and validation data for the use of an innovative error‐corrected sequencing technology known as duplex sequencing (DS) (Kennedy et al. 2014; Salk and Kennedy 2020; Schmitt et al. 2012) to quantify and characterize mutations induced by ROS‐producing agents. DS enables the identification and elimination of sequencing artifacts by individually barcoding each strand of a double‐stranded DNA molecule prior to sequencing, thereby providing an unprecedented level of accuracy for mutation analysis across endogenous loci in the genome (Kennedy et al. 2014). Herein, we explore the utility of DS as a method to quantify mutations in an integrated testing strategy based on our AOP. We apply DS to study the mutation frequency and spectrum induced in TK6 cells following a 24‐h exposure to 4NQO.

## 2 Methods

### 2.1 Cell Culture and Chemicals

TK6 human lymphoblastoid cells were obtained from the American Type Culture Collection (ATCC# CRL‐8015; ATCC, Manassas, VA). Cells were cultured and maintained at 1 × 10^5^ to 1 × 10^6^ cells/mL in RPMI 1640 medium supplemented with 10% heat‐inactivated horse serum, 2 mM of L‐glutamine, and 1 mM of sodium pyruvate, at 37°C and 5% CO_2_. 4‐nitroquinoline‐N‐oxide, ≥ 98% purity, (CAS No: 56–57‐5), purchased from Sigma‐Aldrich, was dissolved and diluted in dimethylsulfoxide (DMSO) (1% v/v), which served as the solvent control. The concentration range selected in this study was initially based on similar experiments using 4NQO (Brüsehafer et al. 2016).

### 2.2 High‐Throughput Single‐Cell Gel Electrophoresis

High‐throughput single‐cell gel electrophoresis, that is, the CometChip assay, was conducted using the Trevigen CometChip 96‐well system (Trevigen, Maryland, United States) with and without the formamidopyrimidine DNA glycosylase (Fpg) enzyme modification to measure oxidative DNA damage and DNA strand breaks, respectively, as per the manufacturer's instructions.

#### 2.2.1 Preparation and Cell Loading

CometChips, which are glass plates covered with agarose gel that contain 30 μm in diameter single‐cell microwells (Trevigen, Maryland, United States), were equilibrated in room temperature phosphate‐buffered saline (PBS), then placed into the 96‐well macrowell former. The cell suspension was filtered through a 40 μM cell strainer to achieve a single‐cell suspension, and the cells were diluted to a concentration of 1.5 × 10^5^ cells/mL. PBS was aspirated from each well, and 100 μL of the cell suspension was added to each well. The plate was covered and incubated at 37°C for 15 min, rocked back and forth a few times, and then incubated at 37°C for another 5 min. Excess cells and culture media were aspirated from all macrowells, then 99 μL of media and 1 μL of treatment (to achieve the concentrations described below) were added to each well.

#### 2.2.2 Chemical Exposure and Fpg Treatment

Cells were treated with a range of 4NQO concentrations (0.016, 0.032, 0.062, 0.125, 0.25, 0.5, or 1 μg/mL) or an equivalent volume of DMSO (1% v/v) for 2, 4, or 6 h. Each treatment was performed in both biological and technical triplicates. Plates were incubated at 37°C for 2, 4, or 6 h. After the chemical exposure, excess media were aspirated, and the surfaces of the CometChips were washed gently with PBS to remove excess, unloaded cells. A layer of molten low melting point (LMP) agarose was applied on the surface of the CometChip to cover the microwells loaded with cells. Chips were then submerged in lysis buffer (Trevigen catalog number: 4250–050‐01, Trevigen, Maryland, United States) and allowed to lyse for 1 h at 4°C under light occlusion. All chips were equilibrated in Fpg enzyme buffer to ensure equal conditions. Chips were submerged in Fpg enzyme reaction buffer (HEPES 40 mM, KCl 0.1 M, EDTA, 0.5 mM KOH, Bovine serum albumin 0.2 mg/mL, pH 7.5) to equilibrate at room temperature for 15 min; this step was repeated twice using fresh buffer. The chips were then placed either in: (a) Fpg enzyme reaction buffer with the Fpg enzyme (1:10,000 dilution, New England Biolabs, Ipswich, Massachusetts); or (b) Fpg enzyme reaction buffer only. Chips were then incubated at 37°C for 20 min and then transferred to cold alkaline electrophoresis buffer (0.2 M NaOH, 0.2 M EDTA, 0.1% TritonX) to stop the enzyme reaction.

#### 2.2.3 Electrophoresis

Chips were rinsed with 1x PBS, then submerged in cold alkaline electrophoresis buffer (0.2 M NaOH, 0.2 M EDTA, 0.1% TritonX) at 4°C for 20 min under light occlusion; this step was repeated with fresh buffer. Chips were then secured in a CometAssay electrophoresis chamber and covered with 700 mL alkaline electrophoresis buffer. Electrophoresis was performed at a constant voltage of 22 V and variable current at 280 mA for 50 min at 4°C. The chips were then removed from the electrophoresis chamber and submerged in the first neutralization buffer (400 mM Tris, pH 7.4) for 15 min at 4°C under light occlusion; this step was repeated using fresh buffer, then chips were submerged in the second neutralization buffer (20 mM Tris, pH 7.4) for 30 min at 4°C under light occlusion.

#### 2.2.4 Imaging and Data Analysis

Prior to imaging, the chips were stained with 0.2X SYBR gold diluted in the second neutralization buffer (20 mM Tris, pH 7.4) overnight. Then, chips were placed in a clean one‐well plate and imaged with the 5X objective of a Leica DMi8 automated confocal fluorescence microscope (Leica Microsystems, Wetzlar, Germany). The resulting TIFF images were analyzed with Trevigen Comet Analysis software (Bio‐Techne, Devens, MA, USA). This software scans each image, identifies analyzable comets, and determines the percentage of DNA in the comet tails by comparing the fluorescence intensity of the tail to the total fluorescence intensity of the comet. The median percent tail DNA of all the comets found in each well was calculated by the software. Each well contained 50–330 analyzable comets.

Technical triplicates from all three experiments were averaged to produce a 1% tail DNA value for each concentration at each time point. Fold changes in % tail DNA in the −Fpg assay were calculated by dividing the average % tail DNA in exposed samples by the average % tail DNA in the solvent control. The fold changes in the +Fpg assay were calculated as follows: [% Tail DNA_(+Fpg)_ − % Tail DNA_(−Fpg)_]_Exposed_/[% Tail DNA_(+Fpg)_ − % Tail DNA_(−Fpg)_]_Control_.

### 2.3 Microflow MN Assay

MN frequencies were quantified using the in vitro MicroFlow MN assay (Litron Laboratories, Rochester, NY, USA) as a proxy for chromosomal aberrations. The cells were exposed to a range of 4NQO concentrations (0.008, 0.016, 0.031, 0.062, 0.125, 0.25, 0.5, or 1 μg/mL) or an equivalent volume of DMSO (1% v/v). Cells were treated in 96‐well plates, at a cell suspension volume of 100 ± 0.1 μL and a density of 2.0 ± 0.25 × 10^5^ cells/mL. Cells were exposed to DMSO or 4NQO for 24 h at 37°C and 5% CO_2_.

The MicroFlow MN assay was performed after a 24‐h exposure. Each treatment was performed in triplicate. After the 24‐h exposure, cells in control wells were counted to confirm that at least 1.5 cell cycles had occurred, harvested, and flow cytometry analyses were conducted following the Litron Labs in vitro MicroFlow kit instruction manual (Avlasevich et al. 2006). Briefly, cells were collected and then dyed with Nucleic Acid Dye A to identify dead or dying cells and incubated for 30 min under a light source. Cells were lysed with the MicroFlow kit's proprietary Complete Lysis Solution 1 and incubated for 1 h in the dark at 37°C. Cells were then lysed with the MicroFlow kit's Complete Lysis Solution 2 and incubated for 30 min in the dark at room temperature. Both Complete Lysis Solutions contain Nucleic Acid Dye B to stain nuclei and micronuclei. Samples were stored in the dark at room temperature for 24 h before the fluorescence of both nucleic acid dyes was read using a Miltenyi Biotec MACSQuant Analyzer 10 flow cytometer with an integrated 96‐well MiniSampler. Instrument settings followed instructions stated in the MicroFlow MicroNucleus Analysis kit (In vitro, 96 well) (Litron Laboratories, Rochester, NY).

### 2.4 Error‐Corrected Sequencing

Mutations were measured using error‐corrected DS (TwinStrand Biosciences, Seattle, WA, USA). The DNA libraries for DS were built using the TwinStrand DuplexSeq Human Mutagenesis kit that uses the TwinStrand v1.0 Human Mutagenesis panel (TwinStrand Biosciences, Washington, United States). We note that the kit is not available for purchase from TwinStrand Biosciences at the time of manuscript publication. The panel consists of 20 target sites that are 2400 base pairs in length, for a total target region of 48 kb. The target sites are distributed across 20 autosomal chromosomes, spanning genic and intergenic regions, as well as coding and noncoding regions, and are representative of the complete genome with respect to the GC content (Valentine III et al. 2020). The target regions were chosen to exclude regions where mapping quality could be compromised (highly repetitive elements or pseudogenes), and genes reported to have a role in cancer (based on the Catalogue of Somatic Mutations in Cancer (COSMIC) database) or under positive selective pressure (Valentine III et al. 2020).

#### 2.4.1 Cell Pellet Preparation and Chemical Exposure

Cells were exposed for 24 h to 4NQO (0.006, 0.008, and 0.016 μg/mL), or an equivalent volume of DMSO (1% v/v) and sampled 24 h later for DS. Each treatment was performed in duplicate and solvent controls were in triplicate. The 4NQO concentration range was chosen according to the relative survival observed in the MN assay; the top concentration, 0.016 μg/mL, induced a 45% decline in relative survival after 24 h, in line with the recommendations for concentration selection in the Organisation for Economic Co‐operation and Development (OECD)'s test guideline for the MN assay (Avlasevich et al. 2006; OECD 2016).

Cell pellets were prepared at Inotiv before being shipped to Health Canada for DS library preparation. Cells were treated in a 12‐well plate, at a cell suspension volume of 3 ± 0.1 mL and a density of 2.0 ± 0.25 × 10^5^ cells/mL. Cells were exposed to DMSO or 4NQO in duplicate for 24 h at 37°C and 5% CO_2_. After the 24‐h exposure, cells were counted to confirm that at least 1.5 cell cycles had occurred. Then, cells were washed with 1X PBS pH 7.4, resuspended in fresh culture medium, and returned to the same incubator for another 24 h. After a total incubation period of 48 h, cells were counted to confirm that at least 1.5 cell cycles had occurred during the recovery period; then cells were washed with 1X PBS, centrifuged, and PBS was removed. The remaining pellet was flash‐frozen in liquid nitrogen and stored at −80°C before being shipped to HC.

#### 2.4.2 DNA Extraction, Library Preparation, and Sequencing

DNA was extracted from the cell pellets using the Qiagen DNeasy blood and tissue kits (Catalog number: 69504, Qiagen, Hilden, Germany) according to the Qiagen user manual. DNA concentration was measured using a Qubit 4 Fluorometer (Invitrogen, Waltham, MA, USA). DNA integrity was measured using an Agilent 2100 Bioanalyzer system (Agilent Technologies Inc., Santa Clara, USA). Samples were confirmed to have a DNA integrity number (DIN) greater than 7, where a DIN of 1 indicates that the DNA is fully degraded and a DIN of 10 indicates that DNA is fully intact based on the dispersion of the DNA band in gel electrophoresis.

Libraries were prepared using TwinStrand's Human Mutagenesis DS Kit according to the manual (Human Mutagenesis Kit TwinStrand Biosciences Inc., Washington, United States) and as previously described (Cho et al. 2023; Wang et al. 2021) with minor modification. Briefly, 500 ng of DNA per sample was fragmented enzymatically to about 300 base pairs (confirmed on an Agilent TapeStation). The ends of the DNA fragments were repaired and A‐tails were added. Illumina adapters and unique molecular identifiers were ligated to the DNA. Labeled DNA was probed with the TwinStrand v1.0 Human Mutagenesis panel and amplified by PCR for target enrichment. Target enrichment was performed twice before the final clean‐up and quantification. DNA concentration was measured using a Qubit 4 Fluorometer (Invitrogen, Waltham, MA, USA). Library size was measured using an Agilent 2100 Bioanalyzer system (Agilent Technologies Inc., Santa Clara, USA). Libraries were then pooled to a final concentration of 10 nM per sample, frozen, and sent to Psomagen (Maryland, United States) for sequencing on a NovaSeq 6000 (Illumina).

#### 2.4.3 Data Interpretation

Sequencing data were uploaded to the DNAnexus platform as demultiplexed FASTQ files and processed through the TwinStrand Biosciences DS Mutagenesis App (Version 3.18.0) (Valentine III et al. 2020). The application processing methods contained in the application were described in detail previously (Valentine III et al. 2020). Briefly, the application determines consensus between the duplex reads based on their unique molecular identifying tags and removes duplicate observations and interspecies contamination. Read pairs were error‐corrected and bases with low quality were masked as “N” for ambiguous base assignment, then duplex consensus reads were created. In order to eliminate biases from double counting bases in overlapping paired‐end reads, the read pairs then went through balanced overlap hard clipping. Variants were called using VarDictJava with all parameters optimized (Lai et al. 2016). The application pipeline produced a summary of sequencing quality metrics, mutation frequencies, mutation spectra, and trinucleotide spectra (Twinstrand Biosciences Inc., Washington, United States). Identical mutations that were identified more than once in the same sample were assumed to have arisen from a single mutational event as described in our previous work (Dodge et al. 2023). Thus, we present a conservative estimate of mutation frequency (sometimes referred to as MFmin), consistent with other studies applying this approach (Cho et al. 2023; Dodge et al. 2023; LeBlanc et al. 2022; Valentine III et al. 2020).

### 2.5 Statistical Analysis

Statistical analyses were conducted in the R environment for Statistical Computing (R Core Team 2021). Analysis of variance (ANOVA) with post hoc Dunnett's test was conducted on MN frequencies using the DunnettTest() function in the DescTools package (Signorell 2024). For the CometChip and DS analyses, generalized linear models were fit to the data using the glm() function assuming an overdispersed binomial error distribution. For the CometChip analysis, the model consisted of the main effects of concentration, time point, and Fpg treatment with all the two‐way and three‐way interactions. For both the CometChip and DS analyses, pairwise comparisons were conducted using the doBy R package (Hojsgaard and Halekoh 2021). The *p* values from the hypothesis tests comparing the percent DNA in tail or MFmin at each concentration to controls were adjusted for multiple testing using the Holm–Sidak correction. For the CometChip analysis, the multiple testing correction was applied within each time point and Fpg treatment independently. The ANOVA table was estimated using the anova() function. The overall concentration effect was tested using the anova() function using the Likelihood ratio statistic. All performed hypothesis tests were two‐sided.

#### 2.5.1 DS Trinucleotide Mutation Spectra and Catalogue of Somatic Mutations in Cancer (COSMIC) Signature Analysis

The trinucleotide mutation spectra of the solvent control and treatment samples were reconstructed using the mutational signatures in the Catalogue of Somatic Mutations in Cancer (COSMIC; version 3.3) using the online application SigProfilerAssignment (URL: https://cancer.sanger.ac.uk/signatures/assignment/) (Alexandrov et al. 2020; Díaz‐Gay et al. 2023). The SigProfilerAssignment tool determines the contribution of COSMIC signatures to mutation spectra by identifying the number of mutations that correspond to each COSMIC signature within the overall spectra. The observed mutations at each concentration were first pooled, and the contribution of each COSMIC signature to the reconstructed solvent control and 4NQO spectra was analyzed.

## 3 Results

### 3.1 Oxidative DNA Damage and DNA Strand Breaks Measured by the Comet Assay

To quantify the extent of DNA strand breaks and Fpg‐sensitive DNA lesions, including oxidative DNA damage, the CometChip assay was performed after 2‐ 4‐, or 6‐h exposure (3 experiments, each containing three technical replicates of every condition) to seven concentrations (0.016—1 μg/mL) of 4NQO alongside concurrently exposed DMSO solvent controls. Cells were exposed in chips in duplicate: one chip was treated with the Fpg enzyme (+Fpg) to convert oxidative DNA damage into additional DNA strand breaks, and the second chip was not Fpg‐treated (‐Fpg) to measure only DNA strand breaks (Figure 1).

**FIGURE 2:**
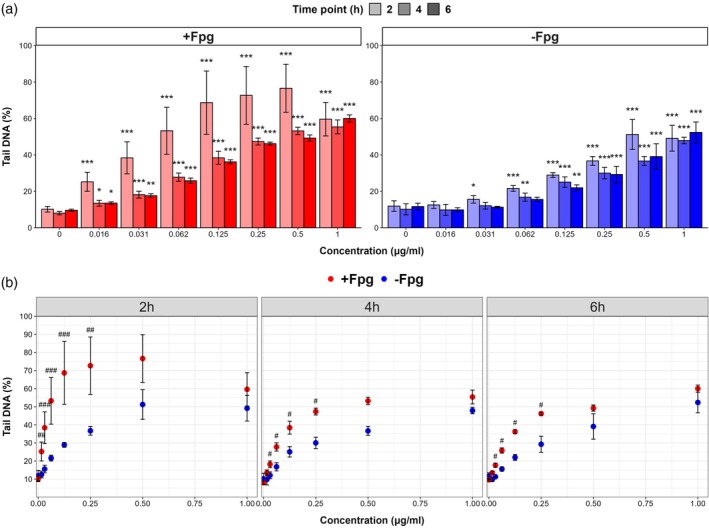
Percent DNA observed in comet tails after 2, 4, or 6 h exposures to 4NQO with Fpg enzyme treatment (in red) and without Fpg enzyme treatment (in blue). A: Each bar represents the average tail DNA (%) of all technical replicates from three experiments (*n* = 3). The error bars represent the standard error of the mean of all replicates. Statistically significant (*p* < 0.05) increases from the solvent control at each time point is indicated by asterisks (*** for *p* < 0.001, ** for *p* < 0.01, and * for *p* < 0.05). B: Each point represents the average tail DNA (%) of all technical replicates from three experiments (*n* = 3). Hashmarks (#) indicate statistically significant differences in tail DNA (%) between the + and −Fpg conditions at the same concentration (### for *p* < 0.001, ## for *p* < 0.01, and # for *p* < 0.05).

First, we established the concentration and temporal effects of 4NQO exposure on % tail DNA measured using the +Fpg CometChip assay. The % tail DNA in this assay reflects oxidative lesions captured as Fpg‐sensitive sites in addition to single and double‐strand breaks (Møller et al. 2018; Owiti et al. 2022). There was a significant increase in the % tail DNA compared to solvent controls starting from the lowest concentration of 4NQO at all three time points using this assay (Figure 2). The % tail DNA increased from the baseline average of 10.0% ± 1.5% to a maximum response of 74.0% ± 11.1% at 0.5 μg/mL 4NQO concentration after 2 h. Statistically significant increases in % tail DNA were also observed at 4 and 6 h for all concentrations tested. However, the levels of % tail DNA following 4NQO exposures at 2 h were significantly higher than the levels at 4 or 6 h at the lowest three concentrations (0.016—0.062 μg/mL 4NQO). There were no differences between % tail DNA levels between 4 and 6 h at any concentration. Overall, the maximal observed responses occurred 2 h postexposure, but % tail DNA remained elevated 6 h postexposure.

We then examined the concentration and temporal effects on the level of DNA strand breaks induced by 4NQO using the −Fpg alkaline CometChip assay. There was a statistically significant increase in DNA strand breaks compared to solvent control starting from 0.031 μg/mL (i.e., the second lowest concentration) at 2 h, and at all three time points by 0.125 μg/mL (i.e., the 4th concentration) (Figure 2). DNA strand break levels increased from an average of 11.0% ± 1.6% tail DNA in solvent controls to an average of 49.2% ± 2.1% tail DNA at the highest concentration (1 μg/mL) across the three time points. There were no statistically significant differences in the DNA strand break levels between time points for any concentration. The results demonstrate a robust concentration‐dependent induction of DNA strand breaks by 4NQO after 2 h of exposure that persists to 6 h of exposure.

To estimate the extent of oxidative and other DNA base damage induced by 4NQO exposure, we compared the +/− Fpg assays at matched time points and concentrations (Figure 2; bottom panels). We observed significantly higher % tail DNA in the +Fpg assay than in the −Fpg assay at 2 h across all concentrations (0.016 μg/mL—0.25 μg/mL 4NQO) except the top two (0.5 and 1 μg/mL). Indeed, 50%–59% of the % tail DNA in the +Fpg assay at the 2‐h time point at 0.016–0.25 μg/mL appeared to be the result of oxidative and Fpg‐sensitive lesions. The largest difference between % tail DNA occurred at 0.125 μg/mL, where there was a 2.2‐fold increase from 28.9% ± 4.3% to 63.2% ± 9.4% tail DNA at 2 h for –Fpg relative to +Fpg. The trend of higher levels of % tail DNA in the +Fpg treatment group was consistent across all time points. Declines in % tail DNA and narrowing margins between median % tail DNA +/− Fpg at 4 and 6 h compared to 2 h postexposure indicate repair of oxidative DNA lesions. Overall, the results demonstrate that 4NQO induces oxidative DNA damage (Fpg‐sensitive sites) that is still observable after 6 h of exposure.

### 3.2 MN Frequency Analysis

**FIGURE 3:**
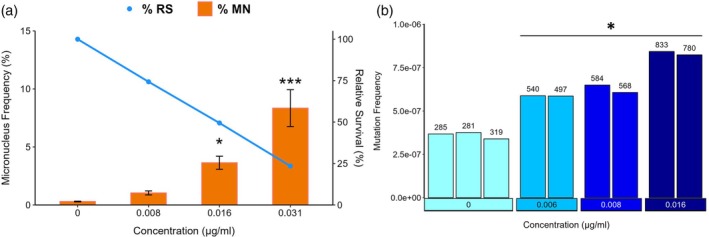
A: Average percentage of micronuclei (orange bars) and relative survival (blue line) observed after a 24‐h exposure to 4NQO (*n* = 3). Error bars represent the standard errors of the mean. Statistically significant increases from the solvent control are indicated by asterisks (*** for *p* < 0.001 and * for *p* < 0.05). B: Mutation frequency per base pair in TK6 cells after exposure to 4NQO or vehicle control (0, DMSO) for 24 h. Cells were sampled 24 h after the end of the 24‐h exposure (i.e., 48 h after the start of the experiment). Each bar represents a replicate and asterisks indicate statistically significant increases from the control (*p* = 0.02 to 1.6e‐7).

The MicroFlow MN assay was performed to measure the percentage of micronuclei observed after a 24‐h 4NQO exposure across an eight‐point concentration range (0.008—1 μg/mL) alongside DMSO controls in triplicate (Figure S1). Note that lower concentrations with respect to the comet assay were used in this analysis as the exposure duration was longer. The relative survival (RS) of the cells declined linearly with increasing 4NQO concentration; % RS declined to 83% of the solvent control at 0.008 μg/mL 4NQO, 55% at 0.016 μg/mL, 25% at 0.031 μg/mL, and to only 10% survival by 0.06 μg/mL 4NQO. The concentrations at and above 0.031 μg/mL achieved higher levels of cytotoxicity than the level recommended by the OECD for the maximum test concentration (i.e., 55% ± 5% cytotoxicity) (OECD 2016); thus, we excluded concentrations above 0.031 μg/mL from further analyses (Figure 3; left panel). There was no significant effect at the lowest concentration, but there was a significant increase in MN frequency at 0.016 and 0.031 μg/mL. The percentage of cells with MN significantly increased from 0.33% ± 0.0003% in the solvent controls to 3.6% ± 0.037% at 0.016 μg/mL (10×; *p* < 0.05).

### 3.3 Mutation Frequency and Spectrum Analysis by DS

DS was applied to quantify the impacts of 4NQO exposure on mutation frequency and spectrum. Mutations were measured in cells after a 24‐h exposure to 4NQO (0.006–0.016 μg/mL) and a 24‐h recovery in fresh media (Figure 3B). The top concentration for the DS study was selected based on the results of relative survival measured in the MN test, which showed that 0.016 μg/ml approached 50% viability. The average number of duplex bases sequenced per sample was ~1 billion, for a total of ~15 billion duplex bases across all samples. The sample with the fewest duplex bases sequenced was 0.75 billion; therefore, all samples met a minimum target of 0.5 billion duplex bases. Mutation frequency was calculated by dividing the number of unique mutant bases by the total number of duplex bases sequenced. We focused our analysis on single nucleotide variant (SNV) mutation frequency and spectrum.

The overall mutation frequency observed in controls was 3.6 × 10^−7^ mutations per bp; after exposure to 4NQO, the overall mutation frequency increased in a concentration‐dependent manner (Figure 3B). All 4NQO concentrations induced a significant increase in mutation frequency compared to solvent controls, reaching a maximum of 2.3‐fold above controls at the highest concentration (8.3 × 10^−7^ mutations per bp).

**FIGURE 4:**
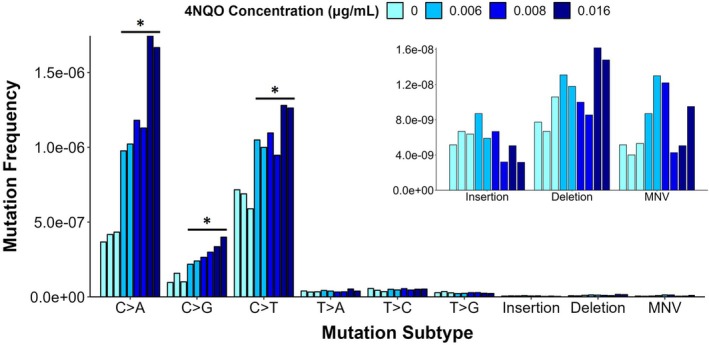
Mutation frequency of each subtype of base substitution (main figure), insertions, deletions, and multinucleotide variants (MNV) (inset figure). Cells were sampled 24 h after the conclusion of the 24‐h exposure (48 h total). Each bar represents a replicate.

In addition to determining mutation frequency, DS enables the characterization of the mutation spectrum. We thus also classified the mutations by base substitution type (Figure 4). C > T followed by C > A mutations were the most prevalent in controls. 4NQO treatment caused concentration‐dependent increases in the frequencies of C > A, C > G, and C > T mutations, with a statistically significant fold increase of 4.2, 3.1, and 1.9 at the highest concentration, respectively. The proportions of C > A and C > G also increased with concentration; the highest concentration induced a 1.8‐ and 1.4‐fold increase in the proportions of C > A and C > G, respectively. (Table 1).

**TABLE 1:** Observed oxidative DNA damage, DNA strand breaks, micronuclei, and mutations summarized as fold change (FC).

Method	Comet + Fpg	Comet + Fpg	Comet + Fpg	Comet −Fpg	Comet −Fpg	Comet −Fpg	Micronucleus frequency	DS
Key event (KE)	Oxidative DNA damage	Oxidative DNA damage	Oxidative DNA damage	DNA strand breaks	DNA strand breaks	DNA strand breaks	Chromosomal aberrations	Mutations
Time point	2 h	4 h	6 h	2 h	4 h	6 h	24 h	24 h + 24 h
FC compared to	DNA strand breaks and control^a^	DNA strand breaks and control^a^	DNA strand breaks and control^a^	Control	Control	Control	Control	Control
Concentration (μg/mL)								
0.006	—	—	—	—	—	—	—	**1.6**
0.008	—	—	—	—	—	—	3.5	**1.7**
0.016	**7.3**	1.7	1.8	1.1	1.0	0.8	**12.1**	**2.3**
0.031	**13.2**	**2.9**	**3.1**	**1.3**	1.7	1.0	27.8^b^	—
0.062	**18.3**	**5.2**	**5.0**	**1.8**	**1.7**	1.3	—	—
0.125	**22.9**	**6.3**	**6.9**	**2.4**	**2.5**	**1.9**	—	—
0.25	**20.8**	**8.3**	**8.2**	**3.1**	**3.0**	**2.5**	—	—
0.5	14.6	7.9	5.0	**4.3**	**3.6**	**3.4**	—	—
1	6.0	3.6	3.7	**4.1**	**4.7**	**4.5**	—	—

*Note:* For Comet −Fpg, Micronucleus frequency, and DS, bold font indicates statistical significance (*p* < 0.05) in comparison to control. However, for Comet +Fpg, bold font indicates statistical significance (*p* < 0.05) in comparison to Comet −Fpg. “—” not included in the experiment or concentration was excluded because of overt cytotoxicity.

^a^ [% Tail DNA_(+Fpg)_ − % Tail DNA_(−Fpg)_]_Exposed_/[% Tail DNA_(+Fpg)_ − % Tail DNA_(−Fpg)_]_Control_.

^b^ Cytotoxicity > 55%.

**FIGURE 5:**
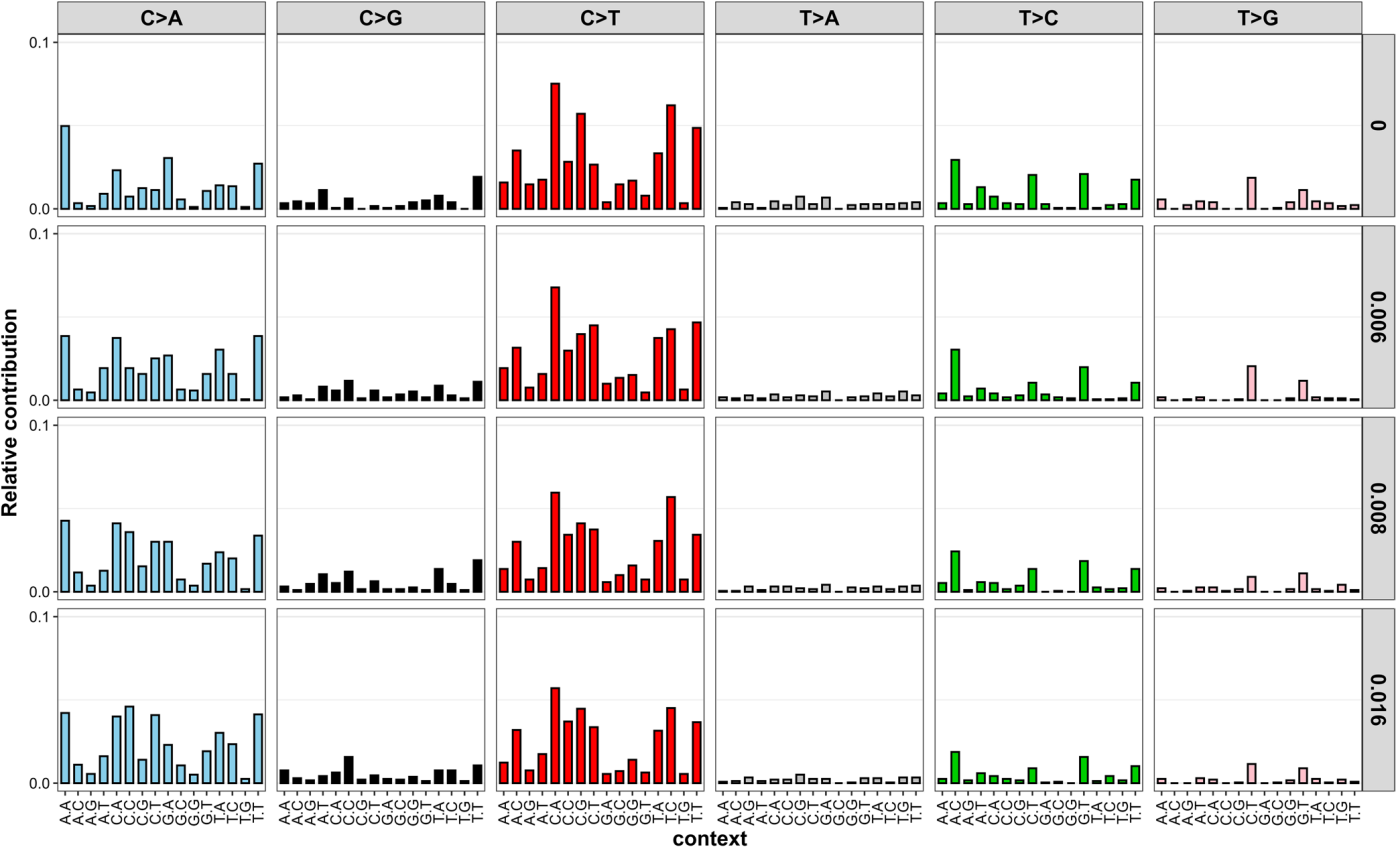
Trinucleotide spectra in TK6 cells after exposure to 4NQO or vehicle control for 24 h. Cells were sampled 24 h after a 24 h exposure (48 h total). Mutation frequencies were averaged for each exposure concentration (*n* = 3 for controls and *n* = 2 for exposed). The substitution subtype is listed at the top, with the two flanking nucleotides shown along the bottom. The Y axis indicates the proportion of each substitution type within the entire population of mutations recovered. Gray bars on the right indicate concentration in μg/mL.

**FIGURE 6:**
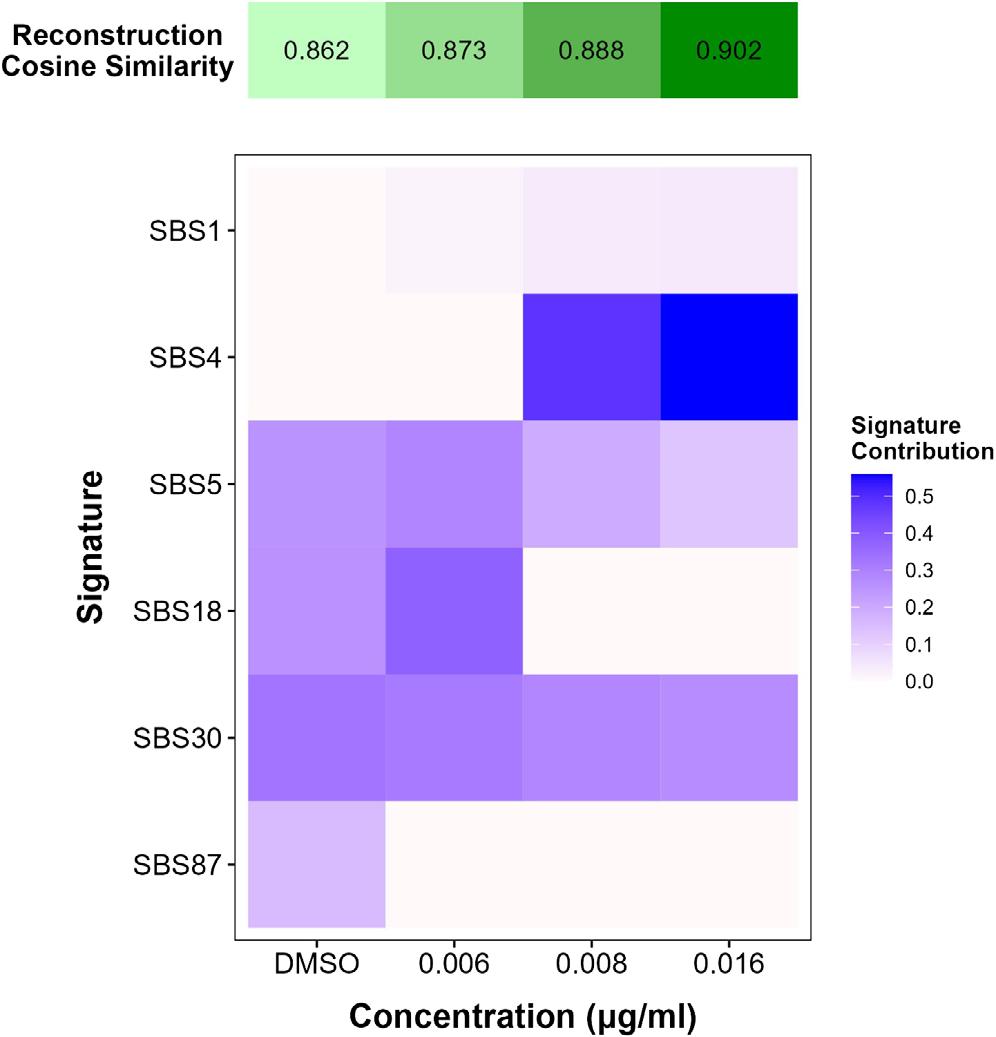
Contribution of COSMIC signatures to the reconstruction of experimental trinucleotide mutation spectra in TK6 cells exposed to DMSO or 4NQO for 24 h, determined using the SigProfiler online application.

Next, we considered the mutation spectrum within the trinucleotide context and conducted signature analyses. The most notable observation here was enrichments in C > A mutations compared to control spectra (Figure 5). We reconstructed the trinucleotide mutation spectra using COSMIC signatures and the SigProfiler online tool to gain insight into the underlying mechanisms driving the spectra in the solvent control and treated samples (Figure 6 and Table 2). COSMIC signature SBS87 (thiopurine chemotherapy treatment) was found to contribute to the DMSO control mutation spectrum and not the 4NQO‐induced mutation spectra. Five signatures contributed to the reconstructions of 4NQO‐induced mutation spectra in TK6 cells: SBS1 (spontaneous or enzymatic deamination of 5‐methylcytosine to thymine), SBS4 (tobacco smoking), SBS5 (clock‐like signature with unknown etiology), SBS18 (reactive oxygen species), and SBS30 (defective base excision repair due to NTHL1 mutation). The contribution of SBS1 and SBS4 to the reconstructed mutation spectra increased with 4NQO concentration, with the largest contribution from SBS4 (0.484–0.56) at the top two concentrations and small contributions from SBS1 (0.018–0.041) at all three concentrations. SBS5 and SBS30 were present at all concentrations with contributions of 0.25–0.13 and 0.33–0.27, respectively; however, the contributions decreased with increasing concentration. SBS18 was observed only in the solvent control (0.26) and at the lowest concentration of 4NQO (0.38).

**TABLE 2:** Contribution of COSMIC signatures to 4NQO mutation spectra reconstruction.

Signature	Proposed etiology^a^	DMSO	0.006 μg/mL	0.008 μg/mL	0.016 μg/mL
SBS1	Deamination of 5‐methylcytosine	0	0.018	0.037	0.041
SBS4	Tobacco smoking	0	0	0.484	0.559
SBS5	Unknown	0.254	0.288	0.193	0.13
SBS18	Reactive oxygen species	0.259	0.379	0	0
SBS30	Defective base excision repair: NTHL1 mutation	0.331	0.316	0.286	0.27
SBS87	Thiopurine chemotherapy treatment	0.156	0	0	0

^a^ Catalogue of Somatic Mutations in Cancer (COSMIC) (https://cancer.sanger.ac.uk/signatures).

## 4 Discussion

An imbalance in intracellular oxidants and antioxidants from exposure to exogenous ROS and oxidants, as well as disturbances in antioxidant defense, can lead to oxidative stress that triggers the events in AOP #296 “Increase in oxidative DNA lesions leading to chromosomal aberrations and mutations” (Cho et al. 2022). This AOP is critical in genetic toxicology as oxidative DNA damage is induced by many xenobiotic exposures. Empirical data required to support the weight of evidence for an AOP and to establish quantitative relationships are currently limited for this AOP. The ideal study design for AOP evaluation involves the use of a single model system that measures multiple KEs from the AOP in the same study to establish dose, temporal, and incidence relationships between the KEs (OECD 2018). Thus, in this study, we used the TK6 cell model to conduct a concentration‐response and time‐series analysis, integrating different methodologies to measure four KEs in AOP #296, using 4NQO as the prototypical oxidative DNA damage‐inducing agent. Our experiment provides an example of an AOP‐informed study design for establishing quantitative associations.

Using the high‐throughput CometChip assay, we demonstrate concentration concordance of the MIE (oxidative DNA damage) and KE2 (Increase, DNA strand breaks) following exposures to 4NQO, as per the Bradford Hill (B‐H) criteria used to evaluate AOPs (OECD 2018). Specifically, we observed a concentration‐dependent increase in Fpg‐sensitive sites, which include both oxidatively damaged bases and DNA strand breaks, starting with the lowest 4NQO concentration after 2 h, but no significant increase in the −Fpg assay until higher concentrations. This is consistent with the B‐H criterion of concentration concordance, where early KEs are detected at lower or equal exposure concentrations than downstream KEs.

The time series analyses applying the +/− Fpg CometChip allowed the assessment of inadequate DNA repair (KE1a and 1b). Specifically, the levels of Fpg‐sensitive sites decreased between 2 and 4 h at all concentrations but did not decrease further between 4 and 6 h. This reduction in Fpg‐sensitive sites suggests that over half of the oxidative lesions occurring at 2 h were repaired by 4 h postexposure, while DNA strand breaks remained mostly unrepaired up to 6 h. In addition, the lack of reduction in the levels of DNA strand breaks or Fpg‐sensitive base lesions between 4 and 6 h indicates that DNA repair was not complete at these time points (i.e., KE1b); this is consistent with previous studies that compared the Fpg‐modified comet assay and the standard comet assay after 3‐ to 4‐h exposures to 4NQO in TK6 and human THP‐1 cells (Azqueta et al. 2013; Møller et al. 2018). Together, the high‐throughput CometChip assay results support the occurrence of KE1 and the quantitative relationship between the MIE and KE2.

Next, we compared the levels of oxidative DNA lesions and strand breaks to mutation frequencies (AO1) and chromosomal aberrations (AO2) to assess concentration concordance between the MIE and AOs. As expected, 4NQO induced an increase in oxidative DNA damage and strand breaks that were detected starting from the earliest time point, supporting the B‐H criterion of temporal concordance of the KER, that is, DNA breaks must be induced for micronuclei and mutations to be formed. While we did not monitor base damage and strand break levels beyond the 6‐h time point, concentration–response in the two AOs indicates inadequate repair of the damage observed at earlier times. Statistically significant increases in MN and mutation frequencies were observed at concentrations that also caused increases in Fpg‐sensitive lesions at all time points, supporting the B‐H criterion of concentration concordance. Additional experiments to quantify the MN frequency in the presence of ROS scavengers or using other methods to modulate the repair of oxidative stress‐induced DNA lesions would be useful to clarify mechanisms of action, and to test the essentiality (another B‐H criterion) of the KE to the AOP.

One challenge in establishing incidence and concentration concordance for KERs is differences in assay sensitivity, dynamic range, and resolution of the methodologies. For example, % tail DNA reflects the total DNA damage occurring in individual single cells, whereas the frequency of individual cells containing MN within a group of cells is represented in the MicroFlow assay. Mutation frequency is quantified at the single base level, with a detection sensitivity of one mutation in 10^7^ bases (Kennedy et al. 2014). The amount of DNA damage required to produce a measurable increase above the baseline varies for each method. Thus, quantitative understanding of the incidence relationship, another B‐H criterion, between the KEs is complicated by the disparate units of measurement for different assays. Quantifying per nucleotide levels of damage could define the KERs more precisely. Methodologies such as high‐performance liquid chromatography [HPLC] (Helbock et al. 1998) and mass spectrometry (Mangal et al. 2009) have been used to quantify specific DNA base lesions including 8‐oxo‐dG. Such a study design would enable direct quantitative associations between the number of oxidative lesions in DNA that are converted into mutations.

Despite the complexities in comparing the endpoints in this study, we explored the quantitative relationship between the response levels for predictive toxicology application (Table 1). In our experiment, we have one concentration in common across all of the assays. Using 0.016 μg/mL 4NQO as a reference point, a statistically significant 7.3‐fold increase in % tail DNA in the +Fpg assay relative to the −Fpg assay and solvent control at 2 h translated to a 12‐fold increase in MN after 24 h and a 2.3‐fold increase in mutation frequency at the 48‐h time point (Table 1). Additional work with more concentrations is clearly necessary to more precisely define the quantitative associations.

A second major objective of our study was to add to the body of literature investigating the use of DS as an effective approach for mutagenicity assessment. Mutation analysis using DS is an emerging methodology in genetic toxicology, and strategies to incorporate this technology in chemical testing are still in development. A previous study explored different recovery periods following a 24‐h exposure to *N‐*ethyl‐*N‐*nitrosourea (ENU) to ensure that cells have undergone sufficient cell divisions prior to DS analysis. Cho et al. (2023) determined that a 24‐h recovery period (48‐h sampling time) was sufficient to detect a concentration‐dependent increase in mutation frequency and that these did not increase with longer sampling times. Thus, the same experimental design was applied in this study. The robust concentration response we observed in mutation frequency supports the suitability of this experimental design for applying DS to analyze 4NQO‐induced mutagenicity in TK6 cells. Additional mutagens with diverse mechanisms must be tested using DS to continue optimizing the methods for its application in vitro.

In addition to mutation frequency, DS provides mutation spectra data to reveal underlying mechanisms of mutagenicity and inform the biological plausibility of KERs. 4NQO and its mutagenic metabolites (e.g., 4‐hydroxyaminoquinoline 1‐oxide (4HAQO)) mainly target guanine bases for adduct formation, including the two most common oxidative guanine lesions, 8‐oxodG and 2,6‐diamino‐4‐hydroxy‐5‐formamidopyrimidine (FapyG), which stably pair with adenine (Bailleul et al. 1989; Cadet and Wagner 2013; Kohda et al. 1991). Thus, increases in C:G mutations (C > A, C > G, C > T) were expected. The largest increase was C > A, which is concordant with previous 4NQO studies using in vitro and in vivo mammalian models (Arima et al. 2006; Ide et al. 2001; Lee et al. 2023; Ryu et al. 1999). However, given the deficient O_6_‐methylguanine DNA methyltransferase (*MGMT*) gene in TK6 cells, a higher accumulation of C:G > T:A is expected in both the baseline and 4NQO‐induced mutation spectra compared to other models (Nagel et al. 2019). Indeed, the proportion of C > T (35%) remained comparable to C > A (45%) even at the highest concentration of 4NQO in TK6 cells; whereas, in MGMT‐proficient mouse models, a smaller proportion of C > T was present relative to C > A (25% and 60%–69% respectively) (Lee et al. 2023; Ryu et al. 1999). These results show that the observed mutation spectrum generated by DS is consistent with what is known about the mutagenic mechanism of 4NQO and the DNA repair status of TK6 cells.

Additional insight into 4NQO's mutagenic mechanisms can be obtained through trinucleotide spectra analysis and signature reconstruction with COSMIC signatures and SigProfiler. These analyses showed that SBS18 (ROS‐induced damage) only appeared in the solvent control and at the lowest 4NQO concentration, while SBS4 (tobacco smoking) contributed the most at the two highest doses. Indeed, 4NQO has been used as an inducer of oral squamous cell carcinoma, a cancer linked to smoking, in rodent cancer studies (Ide et al. 2001). Oxidative stress has also been established as one of the drivers of smoking‐associated DNA damage and carcinogenesis (Kanojia and Vaidya 2006; Kyng et al. 2005; Lee et al. 2023; Miranda et al. 2011). Tobacco smoking (Alexandrov et al. 2013) and related compounds (Kucab et al. 2019) also produce bulky adducts that contribute to mutation spectra rich in C:G > A:T mutation. The absence of a strong SBS18 signature at higher 4NQO concentrations suggests that the dominant mutational process is bulky adduct formation rather than oxidative lesions. However, oxidative stress may still contribute to the overall mutational landscape, particularly at lower concentrations where DNA repair mechanisms may effectively counteract bulky adduct accumulation.

We observed a consistent contribution of SBS30 (NTHL1 deficiency) in all samples (0.33–0.27). NTHL1, along with OGG1, participates in the base excision repair of oxidative DNA damage, including oxidized pyrimidines FapyG and FapyA (Das et al. 2020; Hu et al. 2005). Thus, deficiencies in NTHL1‐mediated DNA repair could theoretically contribute to C > T mutations observed following 4NQO exposure. However, empirical evidence to confirm the NTHL1 status in TK6 cells is unavailable in the current literature. In addition, we must note the caveats of conducting a COSMIC signature analysis on in vitro mutation spectra. The signatures were derived from human cancers, which are likely impacted by multiple factors in addition to chemical exposures in the course of carcinogenesis. Whereas, the mutation spectra were measured after a short‐term exposure with a single mutagen herein. Thus, while the analysis provides insights into potential mutagenic mechanisms in vitro and may be of use for guiding additional studies, more research is needed to define the role of NTHL1‐mediated DNA repair in the observed TK6 mutational signatures.

To summarize, we used an AOP‐informed study design in TK6 cells to examine the qualitative and quantitative relationships between Fpg‐sensitive base damage (including oxidative DNA damage), strand breaks, MN, and mutations. Although our analysis was challenged by different resolutions and sensitivities of the assays, our results suggest that statistically significant increases in DNA strand breaks and oxidative lesions at 2–6 h predict increases in MN and mutation frequencies in TK6 cells following cell division. Alternative models, study designs, methodologies, and additional ROS inducers with different mechanisms should be investigated to continue to broaden the quantitative understanding of the KERs and increase the predictive utility of the AOP. Furthermore, this study demonstrates the application of DS, an emerging technology, using a previously developed experimental scheme for in vitro mutagenicity assessment of oxidative DNA damage in TK6 cells. While additional mutagens must be tested to validate DS and the current study design, the results herein suggest the potential for applying DS as part of an integrated chemical testing approach. Overall, our analysis supports that 4NQO's mutagenic effects are complex, involving both direct DNA adduct formation and oxidative stress. Though bulky adducts appear to be its primary lesions (primarily informed by DS), ROS production during the metabolism of 4NQO provided the opportunity to examine oxidative damage within a controlled experimental system. By integrating multiple assays, we demonstrate that we can partly disentangle these processes and assess their relative contributions to overall genotoxicity.

## Author Contributions

E.H.: conceptualization, investigation, formal analysis, writing – original draft, writing – review and editing. E.C.: formal analysis, visualization, writing – original draft, writing – review and editing. C.D.S.: Investigation, writing – review and editing. A.W.: Formal analysis, writing – review and editing. L.R.: writing – review and editing. J.J.S.: writing – review and editing. F.M.: conceptualization, supervision, funding acquisition, writing – review and editing. C.L.Y.: conceptualization, supervision, funding acquisition, writing – original draft, writing – review and editing.

## Supporting information


**Figure S1.** Average percentage of micronuclei (orange bars) and relative survival (blue line) observed after a 24‐h exposure to 4NQO (*n* = 4). Error bars represent the standard errors of the mean. This figure displays all concentrations tested using the MicroFlow assay, including those that induced overt cytotoxicity (> 55%) that were excluded from further analysis.

## Data Availability

All sequencing data have been uploaded to the National Center for Biotechnology Information's Sequence Read Archive under project number PRJNA1192975 (https://dataview.ncbi.nlm.nih.gov/object/PRJNA1192975?reviewer=4dig33gpv7jf78br4c6jii3m2g).
