# Supplementary material for: Adverse Outcome Pathway‐Informed Integrated Testing to Identify Chemicals Causing Genotoxicity Through Oxidative DNA Damage: Case Study on 4‐Nitroquinoline 1‐Oxide
Source: Environ Mol Mutagen. 2025 May 8;66(4):185–98. doi: 10.1002/em.70011 (PMC12087725; doi:10.1002/em.70011)
Supplement: Supplementary file 1 — Figure S1. Average percentage of micronuclei (orange bars) and relative survival (blue line) observed after a 24‐h exposure to 4NQO (n = 4). Error bars represent the standard errors of the mean. This figure displays all concentrations tested using the MicroFlow assay, including those that induced overt cytotoxicity (> 55%) that were excluded from further analysis. [file EM-66-185-s001.docx]

**
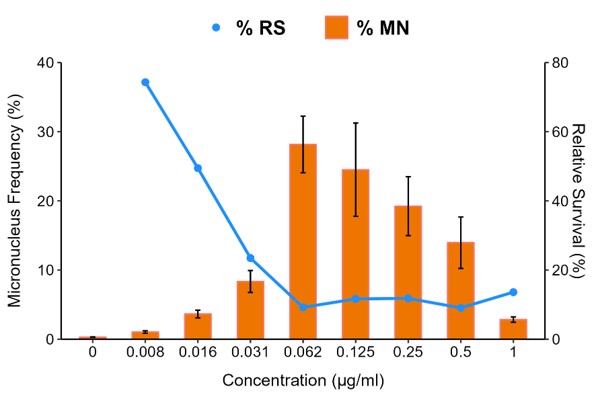
Supplementary Figure S1.** Average percentage of micronuclei (orange bars) and relative survival (blue line) observed after a 24-hour exposure to 4NQO (n = 4). Error bars represent the standard errors of the mean. This figure displays all concentrations tested using the MicroFlow assay, including those that induced overt cytotoxicity (>55%) that were excluded from further analysis.
